# Effect of Drilling Parameters and Tool Geometry on the Thrust Force and Surface Roughness of Aerospace Grade Laminate Composites

**DOI:** 10.3390/mi14071427

**Published:** 2023-07-15

**Authors:** Çağın Bolat, Uçan Karakılınç, Bekir Yalçın, Yahya Öz, Çağlar Yavaş, Berkay Ergene, Ali Ercetin, Fatih Akkoyun

**Affiliations:** 1 Department of Mechanical Engineering, Faculty of Engineering, Samsun University, 55420 Samsun, Turkey; cagin.bolat@samsun.edu.tr; 2 Department of Computer Programming, Vocational School of Technical Science, Isparta Applied Science University, 32200 Isparta, Turkey; ucankarakilinc@isparta.edu.tr; 3 Department of Mechanical Engineering, Faculty of Technology, Afyon Kocatepe University, 03200 Afyonkarahisar, Turkey; 4 Advanced Composite Materials Technology Center, R&D and Technology Directorate, Turkish Aerospace, 06980 Ankara, Turkey; yahya.oz@tai.com.tr; 5 Karcan Cutting Tools, Organized Industrial Site, 26110 Eskişehir, Turkey; c.yavas@karcan.com; 6 Department of Mechanical Engineering, Faculty of Technology, Pamukkale University, 20160 Denizli, Turkey; bergene@pau.edu.tr; 7 Department of Naval Architecture and Marine Engineering, Maritime Faculty, Bandırma Onyedi Eylül University, 10200 Bandırma, Turkey; aercetin@bandirma.edu.tr; 8 Department of Mechanical and Metal Technologies, Trabzon Vocational School, Karadeniz Technical University, 61300 Trabzon, Turkey; fatihakkoyun@ktu.edu.tr

**Keywords:** carbon fiber reinforced polymers, drilling, thrust force, surface roughness, delamination

## Abstract

Carbon fiber-reinforced plastics (CFRPs) have been specially developed to enhance the performance of commercial and military aircraft because of their strength, high stiffness-to-density ratios, and superior physical properties. On the other hand, fasteners and joints of CFRP materials may be weak due to occurring surface roughness and delamination problems during drilling operations. This study’s aim is to investigate the drilling characterization of CFRPs with different drilling parameters and cutting tools. Drilling tests were performed with the Taguchi orthogonal array design (L18: 2^1 3^3). Tests were conducted with three levels of cutting speed (15, 30, 45 m/min), three levels of feed rate (0.05, 0.1, 0.2 mm/rev), two levels of drill diameter (3 and 5 mm), and three different types of drills (two twist drills with a point angle of 138° and 120° and one brad drill). Thrust forces were recorded during drilling tests, and afterwards surface roughness and hole delamination were measured. Obtained results were analyzed with Taguchi and two-way ANOVA. The general tendency was that low cutting speed, high feed rate, and small diameter drill caused an increase in thrust force. Surface roughness decreases with increasing tool diameter, decreasing feed, and cutting speed. Delamination factors of the samples dropped depending on decreasing thrust force levels. Remarkably, it is possible to control the delamination factor values via better surface quality. The brad drill and larger point angle have a negative effect on the drilling quality of CFRPs. According to all results, the cutting speed of 45 m/min and feed rate of 0.05 mm/rev using a type II drill having a 120° point angle and 5 mm diameter (12th trial) and the cutting speed of 30 m/min and feed rate of 0.05 mm/rev using a type II drill having a 120° point angle and 3 mm diameter (2nd trial) were determined as optimum drilling conditions.

## 1. Introduction

In recent years, general concerns over climate change and green nature have reached an important point. Hence, the term sustainability and nature-based innovation endeavors have become attractive subjects both for industrial manufacturers and design engineers. On the other hand, some of the major sectors like the automotive, aerospace, and defense sectors are pioneer industries for this purpose. They can contribute to the clean planet project by reducing fuel/gas emissions. Concordantly, proper material selection/design for a specific construction is a highly critical case that directly influences the average design weight, fuel efficiency, gas emission, and total energy consumption [1,2]. Precisely because of that, fiber-reinforced polymeric laminate composites are notably popular in the aerospace industry due to their unique properties like low density, sufficient specific strength, energy absorption ability, and availability to secondary treatments [3,4].

If actual engineering implementations and the technical literature are taken into consideration, there are many combinations of filler/matrix options for polymer laminates. Product-oriented optimization efforts are still being continued by different research groups. Compared to other alternatives, glass fibers [5,6], carbon fibers [7,8], and basalt fibers [9,10] can be seen as the most examined materials. All these fibers possess their own characteristic advantages such as relatively low density, good flexibility, and sufficient rigidity as well as handicaps like relatively low elastic modulus, high brittleness and relatively high density. At this point, researchers and design engineers usually make decisions in terms of the best combination of performance, usage area, efficiency, and cost. As matrix material, thermoset resins are especially preferred due to their relatively easy molding ability and better mechanical responses.

Considering scholarly work, even though the main investigation topics pile on problems of constituent characterization [11,12], filler/matrix ratio optimization [13,14], improvements of mechanical properties [15,16], manufacturing optimization [17,18], and machining, works on polymer laminate composites have begun to compete with them. Because of the inevitable long-life assembly needs of the automotive and aerospace sectors, drilling operations, which are followed by threading for nuts-and-bolts fitting, are particularly critical for the structural integrity of components. For instance, airplane wing components, fuselage parts, battery boxes of electrical cars, and wind turbine blades can be shown as some of the strategic applications. All these components should be drilled and assembled with minimum delamination, minimum dimensional divergence, and maximum surface quality for the intended service performance. In the process of fabricating holes with precise tolerances, the quality of the hole is vitally important. Factors like delamination, surface roughness, and the diameter of the hole significantly influence this quality. When it comes to drilling fiber-reinforced composites, the primary issue encountered is delamination, which is defined as the splitting of lamina at the entry and exit of the hole. Various common methods are employed to mitigate this problem. These include the manufacture of drills with varied shapes and quality levels, adjusting cutting parameters for optimization, and implementing peck drilling techniques [19,20,21,22,23].

When monitoring recent literature efforts on the drilling features of polymer laminate composites, it is realized that a set of process parameter-focused investigations and tool life optimization works are conducted by various project teams. For instance, Shahri et al. [24] offered a new analytical methodology for a delamination model and focused on the distribution of the drill force on composite laminates. The research group emphasized that critical thrust force levels for delamination could be found via their analytical model. Khashaba et al. [25] carried out a set of drilling tests on glass fiber-reinforced polymer laminates and imparted that cutting force values diminished with ascending cutting speed values due to the temperature-induced softening of the specimens. Upputuri et al. [26] used the fuzzy logic approach to optimize drilling parameters for carbon fiber-added epoxy composites. They put forth that fuzzy logic was a useful instrument for this goal and smaller delamination factor values could be achieved with medium cutting force levels. Yu et al. [27] explored effects of cutting parameters like cutting and advance speed to optimize the drilling of fiber-reinforced resin laminates. The investigation team underlined that simulation-assisted finite element analyses were able to be utilized together with experimental efforts and an increase in the dynamic advance speed would set off an apparent axial cutting force change. Mudhukrishnan et al. [28] concentrated on different tool types during drilling operations of glass fiber-reinforced polypropylene laminates and stated that high-speed steel (HSS) drills led to more thrust forces on account of their reduced hardness and wear inclination in comparison with carbide tools. In another valuable work, Abd-Elwahed et al. [29] applied an artificial neural network approach to foresee drilling properties of woven glass fiber-reinforced epoxy laminates. The survey group indicated that the delamination factor could be kept at a minimum scale with low feed and high cutting speed matching. Lee et al. [30] analyzed the burr formation and tool wear subjects for carbon/aramid fiber-reinforced polymers. They reported that aramid addition resulted in longer tool life and lower thrust forces. Goutham et al. [31] focused on tool wear during drilling of carbon fabric-added epoxy laminates and proclaimed that low feed with high cutting speed was found to be the optimum drilling condition in terms of low thrust force. Khasbaba et al. [32] introduced a novel inexpensive delamination characterization method named as AutoCAD image processing and revealed that this technique had a high accuracy without missing any details in the computed delamination and burr areas compared to other techniques. Babu et al. [33] produced hybrid glass and carbon fiber-reinforced epoxy laminates to explore their drilling performance depending on machining parameters. The researchers pointed out that the feed rate was the least effective parameter whereas the type of drill material was the most contributing variable to cutting force levels. Venkatasudhahar et al. [34] analyzed the impact of the drilling force on the delamination response of kenaf/abaca/carbon fiber reinforced hybrid composites and indicated that high-quality holes were observed with a low feed rate, smaller tool diameter, and medium level cutting speed. Different from these efforts, micro machining characteristics of the laminated composites were also investigated by different researchers [35].

This paper addresses a specific aerospace-grade fiber-reinforced polymer-based composite, and different from the literature, the combined effect of drill geometry (drill diameter and point angle) and main drilling parameters on thrust force levels and surface roughness were examined experimentally. The Taguchi orthogonal array method of L18 was used to determine drilling parameters and the main effect of input variables on thrust force and surface roughness. Moreover, delamination factor calculations, microstructural evaluations, and material removal rate analyses were also performed to discuss the results in detail. The main aim of this paper is to present a promising horizon for real industrial applications and to contribute to assembly and design quality requirements of the target sectors.

## 2. Materials and Methods

### 2.1. Composite Constituents and Sample Manufacturing

In this work, aerospace-grade laminate composites were tested since transportation and defense sectors were adopted as target industries. Therefore, a carbon fiber-reinforced thermoset epoxy matrix composite system was used due to its promising potential for a high strength/weight ratio. Preparation of the composite was performed by uniform hand lay-up of 14 plies of prepregs consisting of unidirectional carbon fibers inside the HexPly^®^ M91 (purchased from the Hexcel Corporation, USA) epoxy matrix. Note that, for instance, such stacking and material are widely used in structural parts of elevators in aircrafts since high toughness, residual compression strength, and glass-transition temperature values are provided by this composite. It is known from scholarly databases that there are various layout styles and fiber orientation patterns for fiber-reinforced laminated polymer composites. Herein, isotropic and anisotropic stacking orders are main variations that were reported in the literature [36,37]. In this study, a unidirectional and isotropic sequence was selected because that kind of order eliminates risks of unpredictable fracture/failure of anisotropic sequences throughout the machining process.

The plies of the composite were vacuum debulked to the mold at 20 °C during the hand lay-up to remove trapped air. Afterwards, autoclave curing was performed. The prepreg stack had a thickness of approximately 3 mm during this step. Autoclave bagging was utilized by placing (1) non-impregnated peel plies below (above the released tool) and on top of the prepreg stack, fixed by a cork, (2) a non-perforated film on the top peel ply and the cork, and (3) two layers of glass fabric on top of the film, with use of a vacuum bag. Curing conditions of the composite were 6.9 ± 0.3 bar as gauge autoclave pressure, 180 ± 5 °C curing temperature, 120 min dwell time and 2 °C/min heat-up and cool down rate. Some physical and mechanical properties of the obtained composite are summarized in Table 1.

### 2.2. Taguchi Experiment Design and Drilling Process Conditions

Researchers need a practical method to determine the effect of variable parameters on target outputs of experimental research, and to determine the experimental conditions and sub-levels. It is a well-known fact that traditional experimental plans are time-consuming and cause high costs and complexity while determining the cause-effect relationship. Taguchi orthogonal arrays were used for the input parameters design determining the number of each experiment condition. This approach was developed and effectively used as a statistical method to optimize various parameters in different levels with a minimal number of experiments in most areas [42,43]. In this study, drilling test parameters for aerospace-grade laminate composite are designed with Taguchi’s L18 (2^1 3^3) orthogonal array, which can be seen in Table 2 and Table 3. Micro drilling tests were conducted with four control parameters; cutting speed with three levels of 15, 30, 45 m/min, feed rate with three levels of 0.05, 0.1, 0.2 mm/rev, tool diameter with two levels of 3, 5 mm, and tool types with twist drill (138°), twist drill (120°), and brad drill (90°). Before the experiments, the runout values of drill tools were measured as approximately 2 µm. In addition, the length/diameter ratio values are 0.97 and 0.58 for the drill diameter of 3 mm and 5 mm, respectively. All drill tools were in a TiAlN coated form (with an average 2.7 µm thickness and 3300 HV). Taguchi experiment outputs are the thrust force and areal surface roughness during the drilling test. Three objective functions commonly used in the selection of the objective function (popularly known as SN ratios) in the Taguchi technique are “smaller the better”, “larger the better”, and “nominal the better” [44]. The objective function selected was “smaller the better” for thrust force and areal surface roughness results in this study.

**Figure 1:**
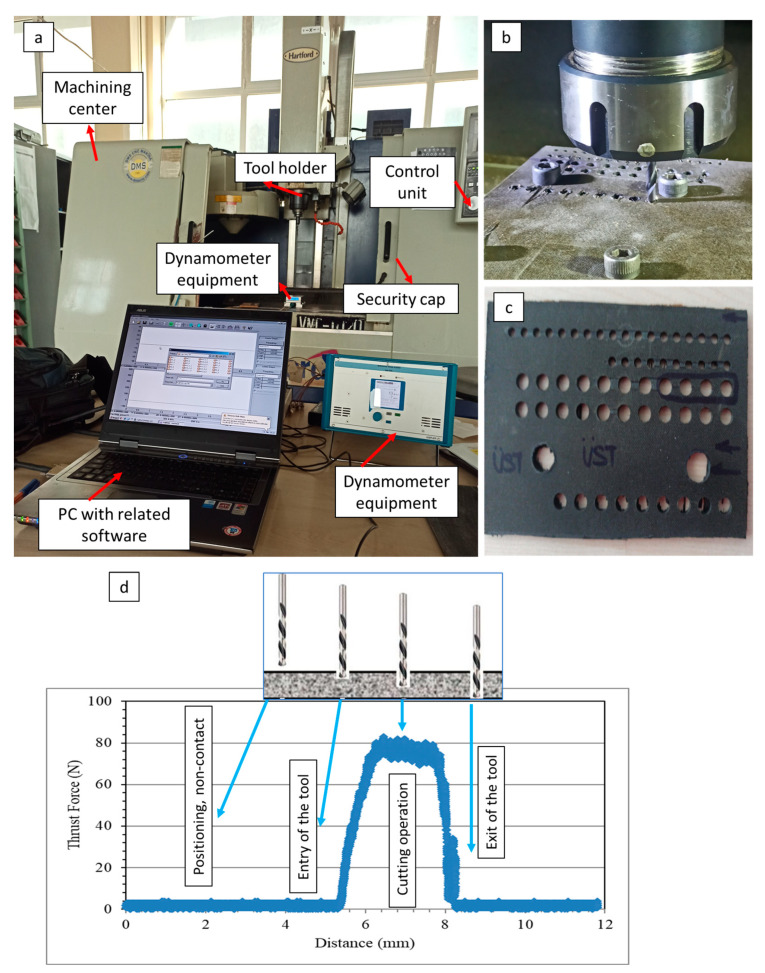
Machining operation: (**a**) real view of the machining center; (**b**) drilling process; (**c**) drilled sample surface; (**d**) an example of the force data acquisition with related interpretations.

**Figure 2:**
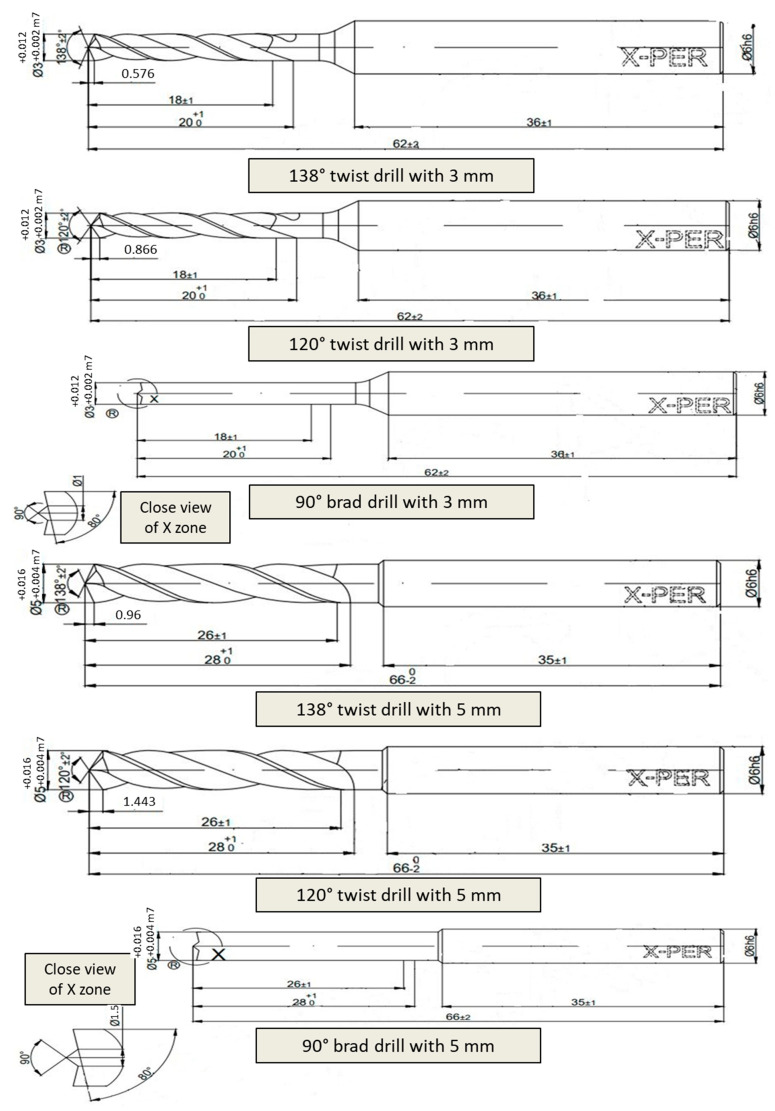
Detailed technical views of utilized drill tools.

Drilling tests were realized by a Hartford VMC-1020 vertical machining center and Karcan WC-6%Co (supplied by Karcan Cutting Tools, Eskişehir, Turkey) drill tools. The thrust force was momentarily measured with a Kistler 9257B multi-component dynamometer (Winterthur, Switzerland) and CutPro^®^ data logger system. Along with the real-time drilling operation image, technical details of the drill tools can be found in Figure 1 and Figure 2, respectively. The areal surface roughness was measured using a Nanovea ST 400 3D optical profilometer (Irvine, CA, USA) from the entrance to the exit of the hole by taking the cross section of the drilled holes. Also, optical analyses were performed with a Nikon SMZ 800 (Tokyo, Japan) microscope using the DpxView software to detect delamination factor values and damage characteristics. To appoint the delamination factor values, the Imaje J software was used, and the ratio of highest diameter to nominal diameter was adopted as emphasized in the literature [28].

## 3. Results and Discussions

### 3.1. Thrust Force Analyses

**Figure 3:**
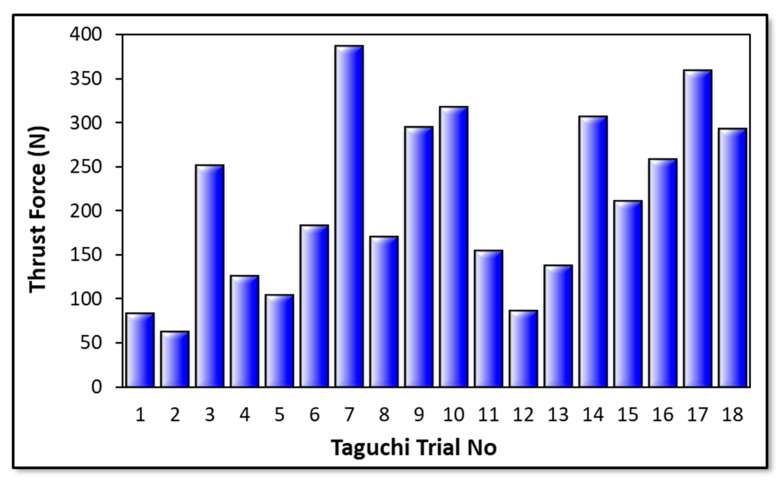
Thrust force results obtained from Taguchi trials.

**Figure 4:**
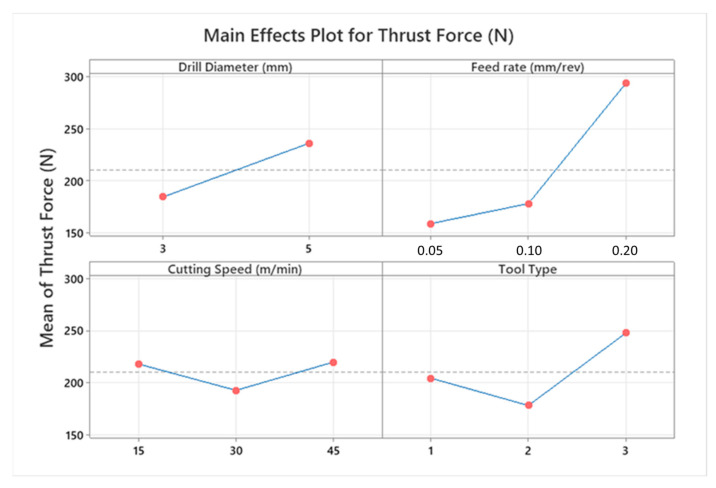
Main effects plot for thrust force levels.

**Figure 5:**
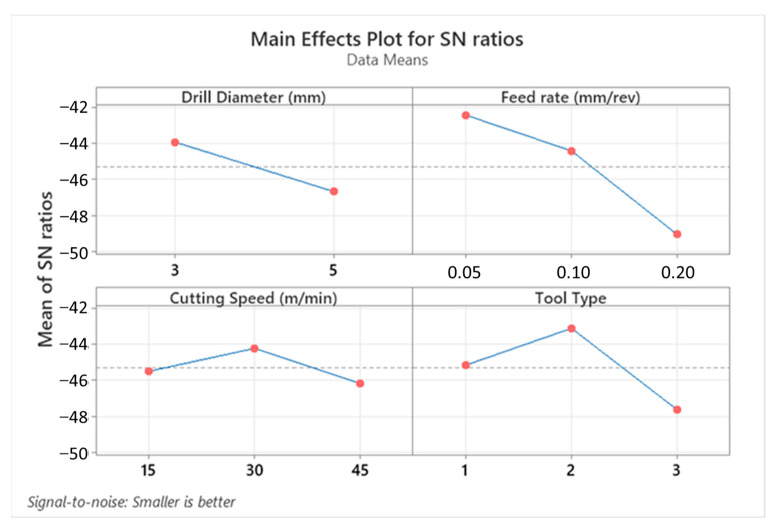
Main effects plot for SN ratios depending on thrust force values.

Cutting force levels are indubitably decisive on the hole quality, tool wear, consumed total energy, and deformation style of the workpiece material during drilling operations of the laminated composites. Thrust force values measured on the laminate components are influenced by cutting process parameters to a great extent. According to the cutting parameters, cutting temperature in polymeric composite drilling is also an important phenomenon with respect to frictional effects between the drill tool and machined composites. In Figure 3, all measured average thrust force values can be found based on the Taguchi L18 design. Furthermore, Figure 4 and Figure 5 show the highlights of Taguchi analyses for each input variable in the point of main effects and signal-to-noise (SN) plots.

According to the outcomes in Figure 4 and Figure 5, it can be propounded that the lowest cutting force levels at an average of 62.2 N are obtained with a smaller drill diameter, lower feed rate, moderate cutting speed, and 120° twist type tool. This combination coincides with the Test 2 in the Taguchi design and can be explained by the thermal softening effect on composite laminates stimulated by the friction-prompter high feed rates and rising contact surface area with increasing tool diameter. Similar observations have been also made by other investigators focusing on the same study subject [44,45]. Since the effect of cutting speed is parallel with temperature-based interactions both on matrix and reinforcement material, that kind of zigzag-style force change is not surprising and has also been noted by other research teams [46]. Based on the actual temperature of the epoxy resin, it is possible to put forward that three characteristic elasticity conditions are seen in the structure, namely the glassy state, rubbery/elastic structure, and viscous-flow style. Concordantly, depending upon the rising cutting temperature, the mechanical response of the matrix resin begins to alter from a glassy state to an elastic state. After the temperature level rises to the glass-transition of the matrix, which is known as approximately 150–200 °C for epoxy [44,47], separation/debonding starts between the fibers and the matrix in combination with an adhesive-style deformation of the polymer on the tool. Therefore, as seen in this work, measured cutting force values can increase with the upper level of the cutting speed that stimulates contact temperatures.

**Figure 6:**
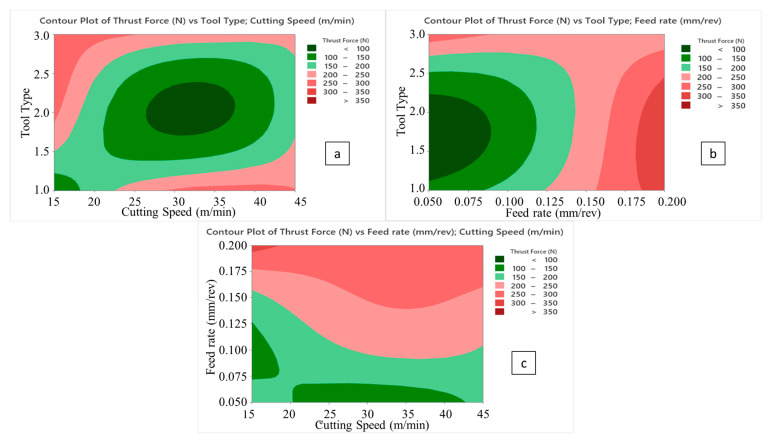
Contour plots of thrust force; tool type/cutting speed (**a**), tool type/feed rate (**b**), and feed rate/cutting speed (**c**).

Laminate samples drilled at lower cutting speeds resist more against deformation and cause more perforating forces because of relatively cold contact surfaces that block the access rigidity decrease on the composites [48,49]. As for the drill tool type, compared to the 138° twist drill, the 120° twist drill possesses a promising capacity to thrust the force drop owing to its superior penetration ability. It is known that an increase in tool point angle also increases the average chip thickness and chip cross section [50]. Besides, during drilling with a traditional twist drill, the chisel edge of the tooltip compresses the laminate specimen aside, particularly at the center zone. As the plastic deformation happens in the chisel edge/workpiece contact area and the drill tool propagates inside the hole, this compression effect continues. Therefore, plastic deformation-based cutting at the chisel edge zone is relatively poor, and there is an indentation-style progress at the drill axis. With the increasing point angle, both the penetration capability of the tool drops, and the compressive effect rises due to the widening contact surface area around the chisel edge. According to the trial sets in the Taguchi design, it is also noted that the highest average thrust force of 386 N belongs to the sample drilled with a cutting speed of 15 m/min and a feed rate of 0.2 mm/rev using a tool point angle of 90° with a brad drill with a 3 mm tool diameter. The reason for this is that chip evacuation becomes more difficult with the softening of the polymer content at low cutting speed and high feed rates in polymeric-based composites. This circumstance is even more evident with the brad drill. It was particularly noted in Figure 6 that the lowest thrust force was obtained in the drilling of the composite with higher cutting speeds and feed rates using a 120° twist drill. Glancing at the previous scientific endeavors, similar findings in which brad drills were subjected to higher thrust forces were reported [47,49]. This phenomenon can be explained by tool design details. Herein, the main deformation/cutting operation of the fiber-reinforced laminate samples proceeds from the chisel edge to the outside cutting edge by utilizing the twist drill. This progress can be defined as a gradual motion. But the main cutting edge contacts the composite with chisel edge through the drilling of CRPCs with the brad drill. This case not only triggers the cutting force rising but also augments the total consumed energy by rising torque values.

**Figure 7:**
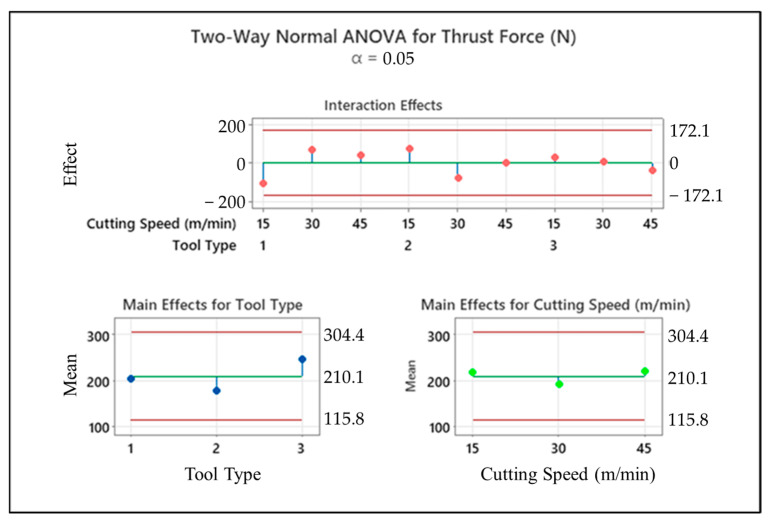
Two-way ANOVA result of thrust force depending on the tool type and cutting speed.

**Figure 8:**
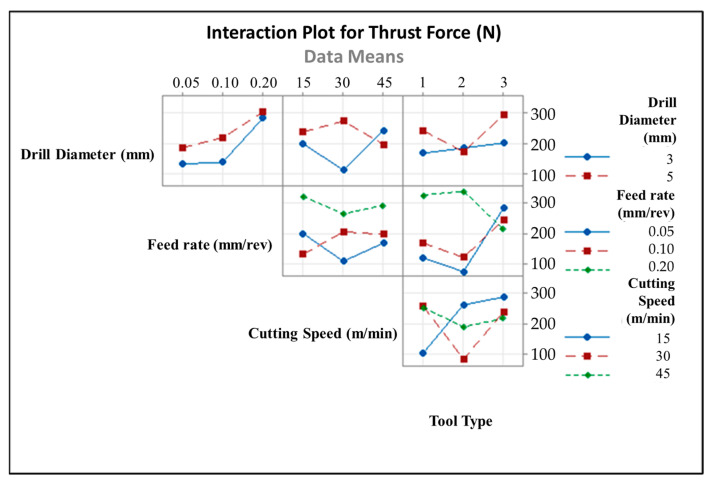
Interaction plot for thrust force values.

Figure 7 illustrates an example of two-way ANOVA results of thrust force values according to cutting speed and tool type. In addition to this, to analyze general results related to the whole interaction couples, Figure 8 depicts interaction plots of all input variables in terms of their effects on the thrust force. It can be deduced from the graphs that the feed rate is the most influential parameter on the thrust force values due to its direct effect on the chip thickness rising and tool type, which is more determinant than the cutting speed. Both for 3 mm and 5 mm tools, especially as the feed rates climb from 0.1 to 0.2 mm/rev, thrust force values go up sharply. In addition, the thrust force showed irregularity and variability when drilling CRPCs with a larger cutting tool; on the other hand, it follows the stable trend for smaller diameters. This case can be attributed to the wider contact surface area that is obtained with bigger drill diameters. At the medium and high level of cutting speeds, a 120° twist tool should be used for low thrust force, but the 138° twist tool may become advantageous for low cutting speeds.

The material removal rate (MRR) is one of the most significant outputs for drilling operations and is highly critical for production duration, part quality, and process planning. In the technical aspect, MRR can be described as the volume of material removed per minute. For the optimum input parameter combination to minimize the thrust force, the average MRR value was calculated. In these studies, the Radwag AS 220.R2 model balance (Radom, Poland) was used, and after measuring the initial and final mass values, the mass change was obtained. Subsequently, the measured mass difference was divided by composite density. Then, the calculated value was proportioned by the time that was recorded during the drilling process. As a result of that, 2314 ± 112 mm^3^/min was found.

Delamination of the composite plies is another issue for the structural integrity and quality of the created hole. In previous studies, different delamination factor formulas are offered by certain investigation groups [51]. In this work, the delamination factor (F_d_) was calculated by using the most common basic approach given with Equation (1), where D_max_ and D_nom_ represent maximum and nominal hole diameter, respectively.

F_d_ = D_max_/D_nom_ (1)

For the optimum input combination to minimize the thrust force, the average delamination factor was also calculated by using optical microscope images and the Image J software. After meticulous measurements, 1.25 was detected as an average delamination factor for the optimum combination, whereas this factor was 1.47 for the worst condition. These outcomes were at an intermediate level and found to be compatible with previous literature efforts focusing on polymer matrix laminate composites. Mohan et al. [52] analyzed the delamination behavior of glass fiber-reinforced polyester resin and noted that delamination factor levels oscillated between 1.040 and 1.006 for optimum cutting parameters. Valega et al. [53] conducted both experimental and numerical analyses to elucidate the delamination mechanisms of glass fiber-added Lapox L-12 resin and put forward that simulation results showed 1.345 while experiments resulted in 0.678 for the delamination factor. Köklü et al. [54] carried out an investigation on the drilling performance of a functionally graded carbon and glass fiber-reinforced epoxy composite. The survey squad noted that delamination factor values escalated with climbing feed rates, and delamination factors as a function of feed varied between 1.05 and 1.45. In another interesting effort, Melentiev et al. [55] revealed that the calculated delamination factor fluctuated between 1.05 and 1.75 based on utilized drill tools as a result of the drilling of carbon fiber-filled polymer laminates.

**Figure 9:**
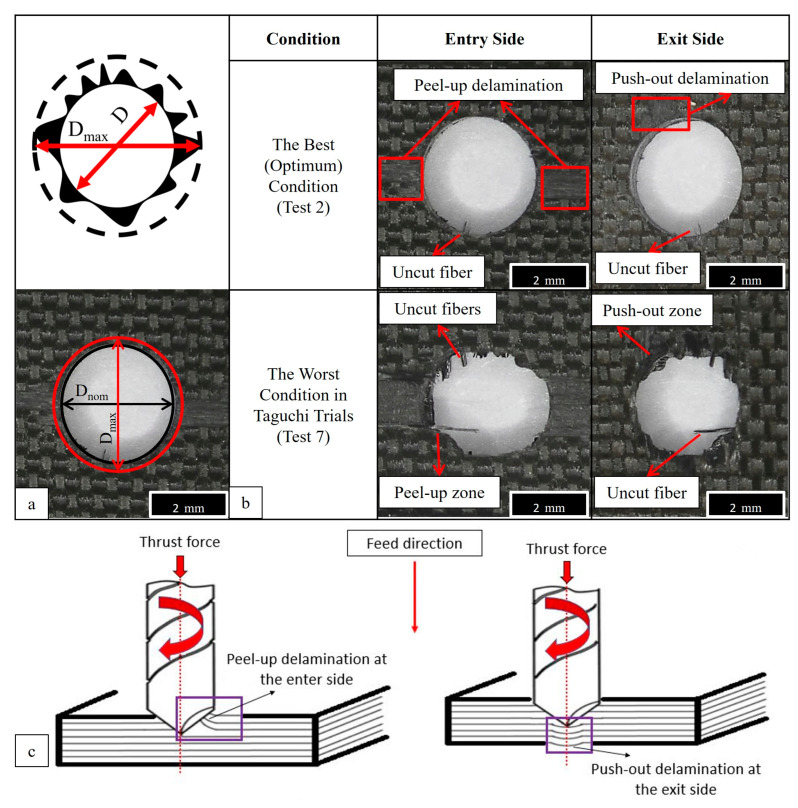
Schematic views of the delamination measurement technique (**a**), deformation-based delamination analyses depending on tool motion (**b**), and schematic views of the delamination types (**c**).

In Figure 9, optical delamination images of the optimal drilling condition and the requirement that led to the highest thrust force level can be monitored. It is widespread knowledge that there are two fundamental deformation/delamination models during the drilling of fiber-reinforced polymer laminates: peel-up delamination (at the entrance side), and push-out delamination (at the exit side). From the optical analyses, it can be deduced that except for a few uncut fibers with negligible peel-up and minor push-out delamination, there are almost flawless holes in the optimized cutting parameters. However, the circumstance is completely different (with major peel-up deformation and apparent push-out zones) for the condition that led to the highest thrust force due to the high feed rate leading to an upper level of deformation force.

### 3.2. Surface Roughness Analyses

**Figure 10:**
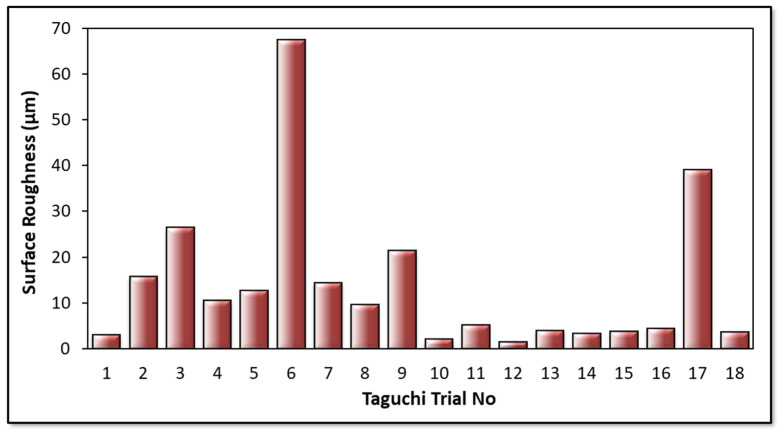
Surface roughness results.

**Figure 11:**
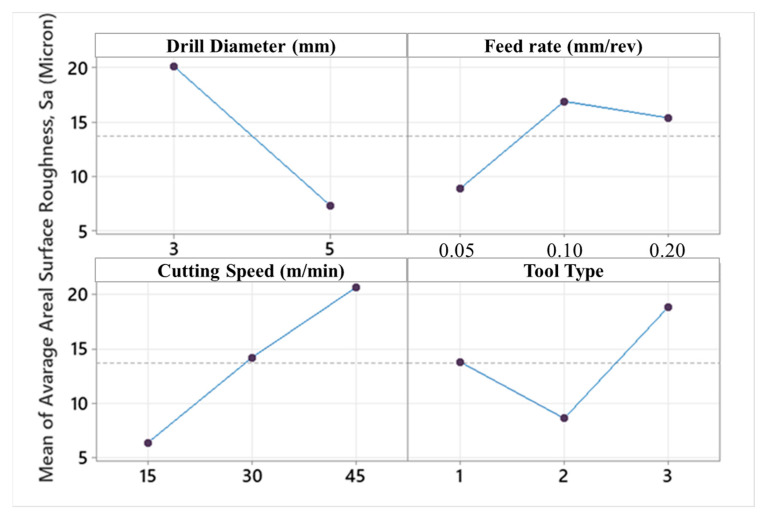
Main effects plot for areal surface roughness values.

**Figure 12:**
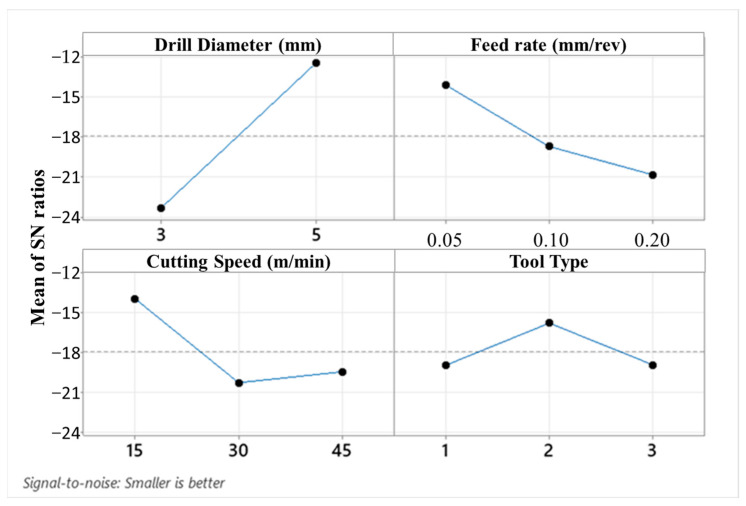
Main effects plot for SN ratios depending on the areal surface roughness values.

Surface roughness is the most important output of cutting parameters and tool performance. It is a factor that affects hole bearing strength, especially in fiber-reinforced composites. Also, surface quality is more critical for special-purpose aerospace-grade laminate composites due to their vital duties in real service conditions. Moreover, average surface roughness values of holes also affect the performance efficiency and mechanical features of the assembly that can be carried out with pins, nuts, bolts, and screws. In Figure 10, surface roughness results of all Taguchi trials are demonstrated. In addition to this, Figure 11 and Figure 12 indicate the areal surface roughness (S_a_) values depending on the Taguchi design results specific to main effects and SN ratio plots.

**Figure 13:**
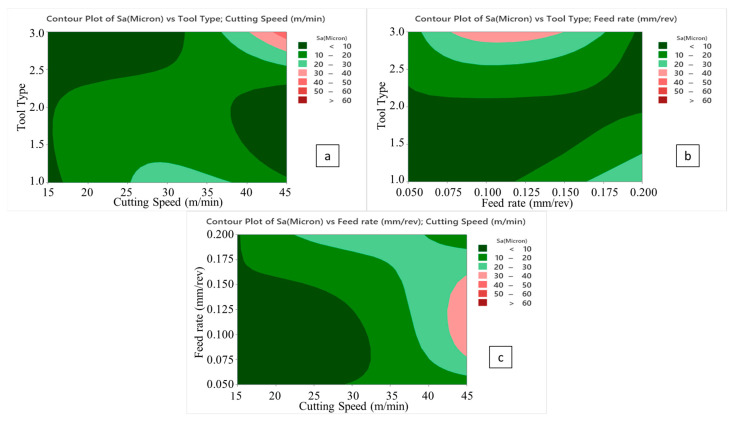
Contour plots of surface roughness; tool type/cutting speed (**a**), tool type/feed rate (**b**), and feed rate/cutting speed (**c**).

Considering Figure 11 and Figure 12, the surface roughness climbs with escalating cutting speed. When the feed rate is higher than 0.5 mm/rev and a brad drill is preferred, the surface quality of the drilled samples becomes more deteriorated. The reason for this is that the evacuation of the chip becomes difficult in drilling with the brad tool. Along with this, the increasing cutting speed also causes difficult chip evacuation and higher cutting temperature that results in bad hole surfaces. In addition to difficulties in chip removal, the presence of thermal softening of the polymer matrix in the hole surface increases the thrust force as well as the delamination factor. Hence, uncut fibers leading to a higher delamination factor also play a crucial role in lowering the surface hole quality. Looking at the experimental data taken from the Taguchi trial sets, the highest average S_a_ value of 67.5 µm is ascertained for the laminate sample drilled with a 3 mm brad type at 45 m/min cutting speed and 0.1 mm/rev feed rate. Nevertheless, it can be asserted that minimum areal surface roughness levels can be reached with a larger drill diameter, lower feed rate, lower cutting speed, and 120° twist drill tool. Typically, as also seen in other efforts [56], the larger feeds tried in this work lead to lesser step numbers and major peak-and-valley structures throughout the drilling direction. For bigger drill diameters, S_a_ values have a decreasing tendency due to the decrease in normal pressure with the widening of the tool rake face. To observe the binary relations in detail, contour diagrams are also shared in Figure 13.

**Figure 14:**
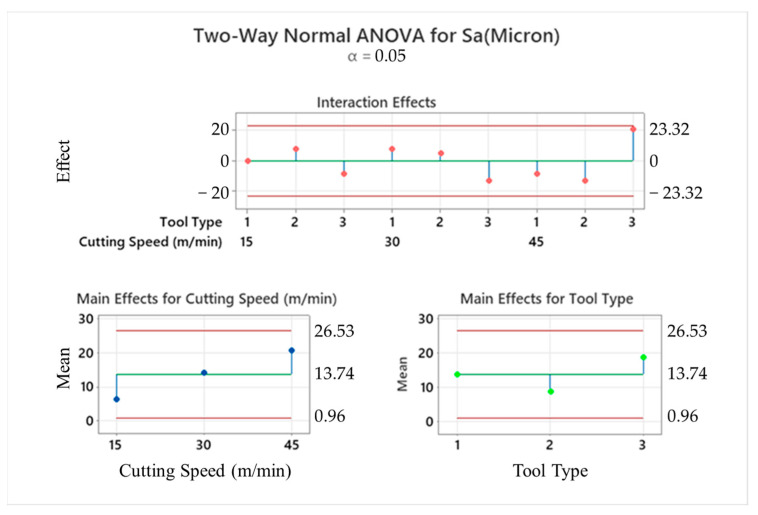
Two-way ANOVA result of surface roughness depending on tool type and cutting speed.

**Figure 15:**
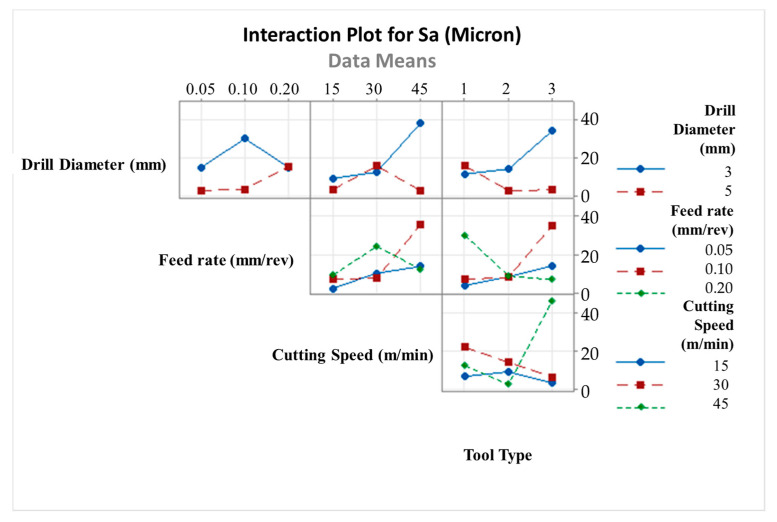
Interaction plot for surface roughness values.

Figure 14 depicts an example of two-way ANOVA results for the surface quality of drilled samples regarding tool type and cutting speed. Along with that, with the intention of exploring effects of all input variables, a comprehensive interaction plot is given in Figure 15. Considering these graphs, it can be seen that cutting speed and drill diameter are the most influential factors on the average S_a_ values of machined laminates. For tools having larger diameters, surface roughness results are prone to fluctuate with augmented cutting speeds, while there is a directly proportional affirmative relationship for tools possessing smaller diameters, especially between medium and high levels. With regard to the feed rate, average roughness values can be diminished with the combination of the lowest feed rate and lowest cutting speed. Also, at higher feed rates, the importance of the drill diameter on the surface quality is reduced on a large scale and the 138° twist drill generates more roughness. Parallel to the cutting speed increment to the top level, the tool having a point angle of 120° comes into the forefront to improve the surface quality, whereas the brad-type tool gives rise to worse surface finishes at the highest speed.

**Figure 16:**
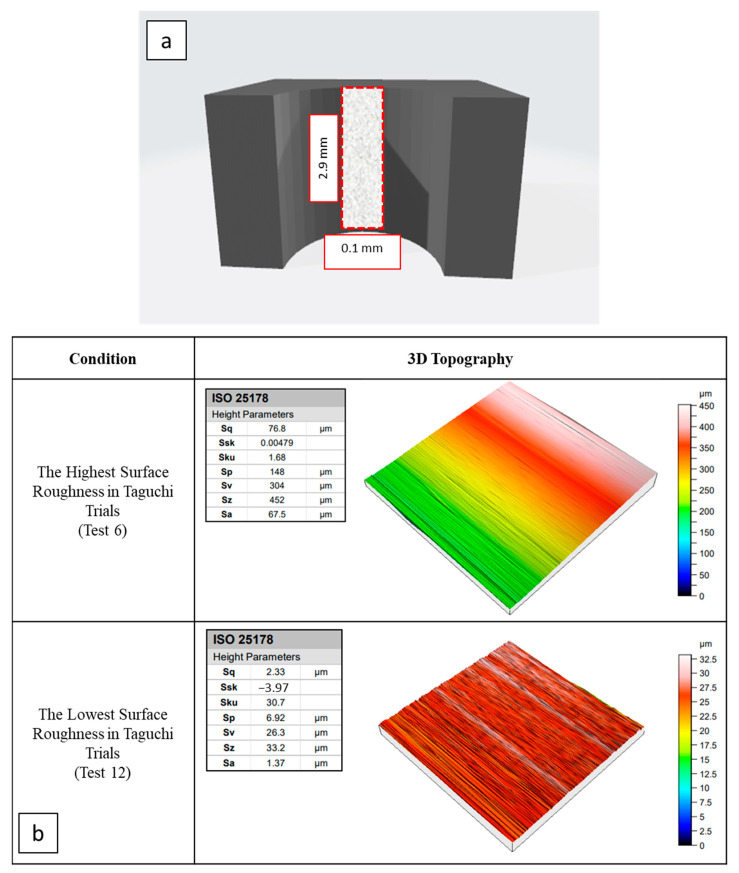
Schematic view of the measured area for areal surface roughness (**a**), and 3D topology views of selected samples (**b**) (ISO 25178 [57]).

In Figure 16, 3D topology results of some significant samples are presented. At this point, samples exhibiting the worst and best surface quality in Taguchi sets were selected. On the other hand, Figure 16 demonstrates that delamination-oriented discontinuity is present at the drill entrance and exit zones for all samples. These delamination defects cause deterioration of the hole surface quality.

**Figure 17:**
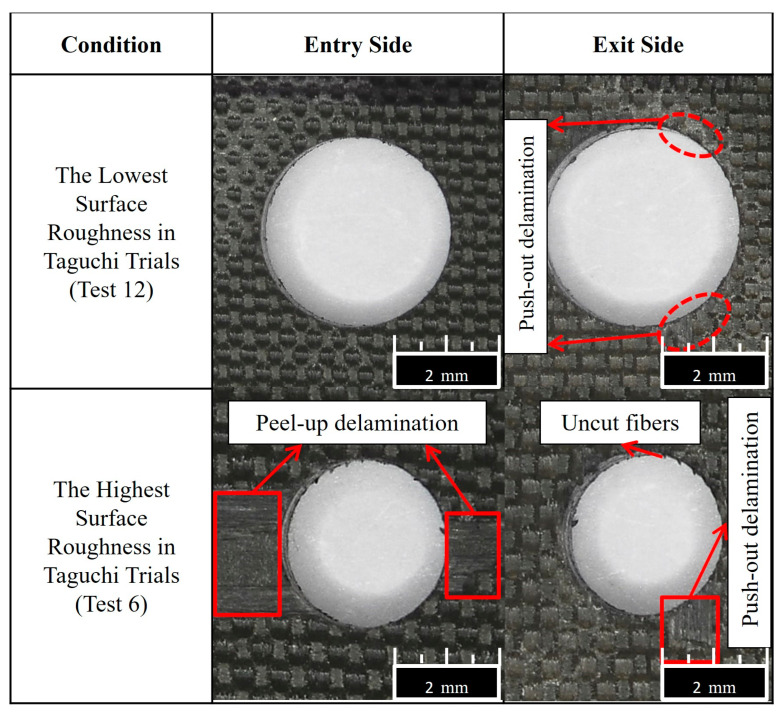
Delamination analyses of drilled samples in terms of surface quality.

If delamination factors of selected samples are compared to each other, it can be understood that the measured delamination factor of 1.22 belongs to the sample possessing the lowest roughness. However, the value of 1.34 is ascertained for the sample having the highest surface roughness. Furthermore, optical analyses of the samples are illustrated in Figure 17. Here, the highest roughness value is seen for the sample drilled with brad-type drill (Test 6). This case can be explained by the high delamination potential of this kind of drill tool. Similar observations were also reported in the literature [58] while the delamination risk of the brad-type drill was noted.

## 4. Conclusions

This experimental study focuses on the drilling performance of an aerospace CFRP composite with different cutting conditions. Thrust force and surface roughness are significantly influenced by cutting speed, feed rate, and the type of drill tool utilized. In particular, the feed rate was found to be the most influential parameter on the thrust force values due to its direct effect on the chip thickness rising and tool type. According to obtained results, the following major findings can be presented:

➢ It is known that chip flow becomes more difficult with the softening of the polymer content at low cutting speed and high feed rates in polymeric-based composites. Therefore, the higher thrust force was measured with a cutting speed of 15 m/min and a feed rate of 0.2 mm/rev using a 5 mm tool diameter.
➢ Brad tool design of type III caused a further increase in thrust force, areal surface roughness and delamination. On the other hand, the type II drill with a 120° point angle has an advantage on cutting force, surface roughness, and delamination.
➢ Areal surface roughness rose with increasing feed rate, cutting speed, and point angle. In addition, an increase in drill diameter caused a diminishment in surface roughness and an increase in thrust force.
➢ Finally, from Taguchi analyses and measurements, the 2nd and 12th drilling conditions can be evaluated as reasonable for low drilling force, areal surface roughness, and delamination.

## Figures and Tables

**Table 1:** Properties of the composite material.

Name	Property	Standard
Resin content (%)	40	-
Composite thickness (mm)	2.92	-
Nominal ply thickness (mm)	0.184	-
Laminate density (kg/m^3^)	1570	-
Glass-transition temperature (°C)	190	EN 6032 [38]
Tensile strength (MPa)	3520	ASTM D3039 [39]
Tensile modulus (GPa)	176	ASTM D3039 [39]
Compression strength (MPa)	1880	ASTM D695 [40]
Compression modulus (GPa)	156	ASTM D695 [40]
Interlaminar shear strength (MPa)	105	EN 2563 [41]

**Table 2:** Drilling parameters and levels.

Symbols	Drilling Parameters	Levels		
		1	2	3
A	Drill diameter (mm)	3	5	
B	Feed rate (mm/rev)	0.05	0.1	0.2
C	Cutting speed (m/min)	15	30	45
D	Tool type (Point Angle)	Twist drill (138°)	Twist drill (120°)	Twist drill (90°)

**Table 3:** Taguchi Orthogonal Array Design (L18 (2^1 3^3)).

Trial No	Tool Diameter (mm)	Feed Rate (mm/rev)	Cutting Speed (m/min)	Tool Type
1	3	0.05	15	1
2	3	0.05	30	2
3	3	0.05	45	3
4	3	0.10	15	1
5	3	0.10	30	2
6	3	0.10	45	3
7	3	0.20	15	2
8	3	0.20	30	3
9	3	0.20	45	1
10	5	0.05	15	3
11	5	0.05	30	1
12	5	0.05	45	2
13	5	0.10	15	2
14	5	0.10	30	3
15	5	0.10	45	1
16	5	0.20	15	3
17	5	0.20	30	1
18	5	0.20	45	2

## Data Availability

Not applicable.
